# A Novel Ultrasensitive ECL Sensor for DNA Detection Based on Nicking Endonuclease-Assisted Target Recycling Amplification, Rolling Circle Amplification and Hemin/G-Quadruplex

**DOI:** 10.3390/s150202629

**Published:** 2015-01-26

**Authors:** Fukang Luo, Guimin Xiang, Xiaoyun Pu, Juanchun Yu, Ming Chen, Guohui Chen

**Affiliations:** Department of Clinical Laboratory, Xinqiao Hospital, Third Military Medical University, Chongqing 400037, China; E-Mails: luofukang@tmmu.edu.cn (F.L.); guimin_xiang@sina.cn (G.X.); yxyxyx_668@163.com (J.Y.); mingchen323817@163.com (M.C.); guohui_chen@sina.cn (G.C.)

**Keywords:** target recycling amplification (TRA), rolling circle amplification (RCA), hemin/G-quadruplex, Nt.BbvCI, ECL

## Abstract

In this study, we describe a novel universal and highly sensitive strategy for the electrochemiluminescent (ECL) detection of sequence specific DNA at the aM level based on Nt.BbvCI (a nicking endonuclease)-assisted target recycling amplification (TRA), rolling circle amplification (RCA) and hemin/G-quadruplex. The target DNAs can hybridize with self-assembled capture probes and assistant probes to form “Y” junction structures on the electrode surface, thus triggering the execution of a TRA reaction with the aid of Nt.BbvCI. Then, the RCA reaction and the addition of hemin result in the production of numerous hemin/G-quadruplex, which consume the dissolved oxygen in the detection buffer and result in a significant ECL quenching effect toward the O_2_/S_2_O_8_^2−^ system. The proposed strategy combines the amplification ability of TRA, RCA and the inherent high sensitivity of the ECL technique, thus enabling low aM (3.8 aM) detection for sequence-specific DNA and a wide linear range from 10.0 aM to 1.0 pM. At the same time, this novel strategy shows high selectivity against single-base mismatch sequences, which makes our novel universal and highly sensitive method a powerful addition to specific DNA sequence detection.

## 1. Introduction

The detection of trace amounts of sequence specific DNA has numerous important applications in clinical diagnosis [1], food safety [2] and environmental monitoring [3]. Many sensitive and selective PCR-based methods have been established in past decade, including real-time fluorescence quantitative polymerase chain reaction (rt-PCR) [4–6], gene chip [7], and gene sequencing [8]. These techniques exhibit the advantages of excellent sensitivity and rapidity, but the sophisticated, expensive equipment and professional skills needed limit their practical application. ECL is the process whereby species generated at electrodes undergo high-energy electron-transfer reactions to form excited states which will generate light. At present, ECL has become a widely used analytical technique in the areas of immunoassay, food testing, and biowarfare agent detection. Thus, many ECL DNA sensors have been proposed as alternatives to PCR-based methods for their simplicity, precise, selectivity, relative low cost and wide dynamic range except sensitivity. ECL DNA sensors typically employ various amplification techniques such as enzymes [9], DNA biobarcodes [10] and nanomaterials [11] to increase sensitivity; however, the sensitivity of such sensorsis not satisfactory. The recently developed rolling circle amplification(RCA) [12–14] and target DNA recycling amplification (TRA) [15–17] techniques demonstrate some improvement in sensitivity; thus, they are widely used in ECL DNA sensor fabrication [18,19].

RCA is an isothermal amplification process mediated by certain DNA polymerases (e.g., Phi29 DNA polymerase), which can produce thousands of repeated sequences and lead to significant signal amplification with speed, high efficiency, and specificity [20]. Therefore, RCA has been widely applied for the highly sensitive detection of DNA [21], RNA [22], and protein [23]. Recently, Ge *et al.* developed a strategy that combined RCA with fluorescent *in situ* hybridization (FISH) and successfully detected microRNAs in tumor cells [24]. The TRA method amplifies signals via cyclical hybridization and digestion with the aid of nucleases such as exonuclease [25], polymerase [26] and nicking endonuclease [27]. DNA nicking endonucleases belong to a special restriction endonuclease family, which can recognize double-stranded DNA and hydrolyze only one objective strand [28]. Nicking endonuclease-assisted TRA has received particular interest due to the virtue its manipulability and isothermal reaction condition. Xu *et al.* have demonstrated colorimetric DNA detection through the use of nicking endonuclease (Nt.A1wI)-assisted TRA, and this system provides a colorimetric detection limit of 0.5 fM within several hours for single-stranded oligonucleotides [29].

Herein, we report a novel highly sensitive strategy for ECL detection of DNA sequences using the *S. aureus* nuc gene (position 894755–894776, GI:47208328), which is highly conserved in all *S. aureus* strains, as a model target [30–32]. Our approach relies on the coupling of Nt.BbvCI-assisted TRA with the RCA reaction for signal amplification and then the formation of hemin/G-quadruplex, which is formed by the intercalation of hemin into a guanine-rich nucleic acid sequence [33]. Hemin demonstrate a significant ECL quenching effect toward the O_2_/S_2_O_8_^2−^ system because the hemin will absorb the oxygen dissolved in the detection buffer like the haemoglobin will absorb the oxygen in blood. Due to Nt.BbvCI-assisted TRA, each target DNA can be used multiple times, leading to the production of numerous intermediate DNA fragments, which can bind specifically designed padlock probes, thus facilitating the RCA reaction and generation of massive hemin/G-quadruplex. In this manner, a considerable quenching effect toward the O_2_/S_2_O_8_^2−^ system is achieved. The integration of these two signal amplification routes and hemin/G-quadruplex in one assay protocol makes it possible to detect an ultralow level of sequence-specific DNA targets.

## 2. Experimental Section

### 2.1. Chemicals and Materials

Nt.BbvCI, NEB buffer 4 (a buffer for Nt.BbvCI reaction), T4 DNA ligase, Phi29 polymerase, dinucleotide triphosphate (dNTP), and bovine serum albumin (BSA) were purchased from New England Biolabs^®^, Inc. (Beverly, MA, USA). 1-Hexanethiol (HT) and Hexaamineruthenium (III) chloride ([Ru(NH_3_)_6_]Cl_3_; RuHex) were purchased from Sigma-Aldrich (St. Louis, MO, USA). Buffer solutions were prepared in our laboratory. A 10.0 mM phosphate buffer solution (PBS, pH 7.4) was used for electrochemical detection and washing. The ECL measurements were performed in 10.0 mM PBS (pH 7.4) containing 50.0 mM K_2_S_2_O_8_ and 0.1 M KCl. A 1× Tris/Borate/EDTA (TBE) buffer comprised 10.0 mM Tris-borate and 1.0 mM EDTA (pH 8.0). All synthetic oligonucleotides were ordered from Takara Biotechnology Co. Ltd. (Dalian, China) and used without further purification. The oligonucleotide sequences used are listed in Table 1.

All other reagents were of analytical grade and used as received. Aqueous solutions were prepared using ultrapure water (specific resistance of 18.0 MΩ-cm).

### 2.2. Gel Electrophoresis and Differential Pulse Voltammetry (DPV)

Different mixtures of SH-CP, target DNA, AP and Nt.BbvCI endonuclease were incubated in reaction buffer (1× NEB Buffer 4) for 1 h at 37 °C. A 15% polyacrylamide gel was prepared by mixing 10.0 mL 30% bis-acrylamide, 140.0 μL 10% ammonium persulfate and 10.0 μL TEMED in 20.0 mL 1× TBE. Then, 15.0 mL of freshly prepared polyacrylamide gel was transferred to sandwich clamps allowed to solidify for 60 min, and 8.0 μL of the reaction product was then loaded into the gel. Gel electrophoresis was performed with a DYY-8C electrophoretic apparatus (Beijing WoDeLife Sciences Instrument Co., Ltd., Beijing, China). The gel was run at 100 V for 1.5 h, stained with ethidium bromide (EB), visualized under UV light and finally photographed with a digital camera. Different modified gold electrodes (AuEs) (a) HT/SH-CP/AuE; (b) Nt.BbvCI/target DNA/AP/HT/SH-CP/AuE; (c) target DNA/AP/HT/SH-CP/AuE (1.0 μM AP, 10.0 fM target DNA and 5.0 U Nt.BbvCI) incubated in 10.0 mM Tris-HCl containing 10.0 mM RuHex (pH 7.4) for 1 h followed by DPV detection with a pulse amplitude of 50 mV and a pulse width of 0.05 s, respectively. DPV is used for electrochemical measurements widely, which can be considered as a derivative of linear sweep voltammetry or staircase voltammetry, with a series of regular voltage pulses superimposed on the potential linear sweep or stairsteps. The current is measured immediately before each potential change, and the current difference is plotted as a function of potential. Therefore, DPV can decrease the interference of impurity, improve sensitivity and detection limit.

### 2.3. Pretreatment of AuE

AuEs were first immersed in a freshly prepared mix of concentrated sulfuric acid and 30% peroxide solution (3:1 (v/v)) for 30 min. Then, the electrodes were thoroughly rinsed with ultrapure water, polished with a 0.3 and 0.05 μm aluminum slurry and sequentially sonicated in ultrapure water, ethanol and ultrapure water for 5 min each. Afterward, the electrodes were electrochemically cleaned in 0.5 M H_2_SO_4_ with potential scanning from −0.3 to 1.55 V until a remarkable voltammetric peak was obtained, followed by sonication and drying with N_2_.

### 2.4. Fabrication of Sensors

A 10.0 μL SH-CP (1.0 μM) droplet was cast onto a pretreated electrode and incubated overnight at room temperature. The electrode surface was then rinsed with PBS and blocked with 1.0 mM HT for 2 h. After washing with PBS, the modified electrode was soaked in 10.0 μL 1× NEB buffer 4 containing 1.0 μM AP, 5.0 U Nt.BbvCI endonuclease and target DNA at various concentrations for TRA at 37 °C for 1 h. Next, the prepared electrodes were rinsed with PBS, dried with N_2_, and a ligation reaction was performed with 1.0 μM padlock probe and 10.0 U T4 DNA ligase in 1× ligation buffer at room temperature for 1 h. Afterward, a 10.0 μL RCA reaction mixture droplet (5.0 U phi29 polymerase, 0.5 mM dNTP and 0.2 mg·mL^−1^ BSA in 1× amplification buffer) was dropped onto the modified electrode and incubated for 2 h at 30 °C. After rinsing with PBS, a 10.0 μL 1.0 mM hemin droplet was cast onto the modified electrode and incubated for 1 h at 37 °C. The ECL intensity of the resulting functionalized electrode was recorded in 10.0 mM PBS (pH 7.4) containing 50.0 mM K_2_S_2_O_8_ and 0.1 M KCl with the scan ranging from 0 to −2 V (*versus* Ag/AgCl) at a scan rate of 50 mV·s^−1^. The voltage of the photomultiplier tube (PMT) was set to 800 V during the detection process.

### 2.5. EC Characterizations and ECL Measurements

Cyclic voltammetry (CV) and DPV were recorded on a CHI 852C electrochemistry workstation (CH Instruments Inc., Shanghai, China). A conventional three-electrode configuration was used for measurement with a modified gold working electrode (4 mm in diameter, CH Instruments Inc., Shanghai, China), a platinum wire counter electrode and an Ag/AgCl (3.0 M KCl) reference electrode. The ECL signal was recorded with a MPI-A electrochemiluminescence analyzer (Xi'an Remax Electronic Science & Technology Co. Ltd., Xi'an, China). CV was performed in 0.1 M KCl solution containing 1.0 mM Fe(CN)_6_^3−/4−^ within the potential range from −0.2 V to 0.6 V under a scan rate of 0.05 V·s^−1^. DPV was performed in 10.0 mM PBS (pH 7.4) between the potential range from −0.5 to −0.1 V with pulse amplitude of 50 mV and a pulse width of 0.05 s, respectively. Before the DPV measurements, the working solution was thoroughly purged with high purity nitrogen for 30 min to avoid the interference from the reduction of oxygen.

## 3. Results and Discussion

### 3.1. Principle of the Sensing System

**Figure 1.**
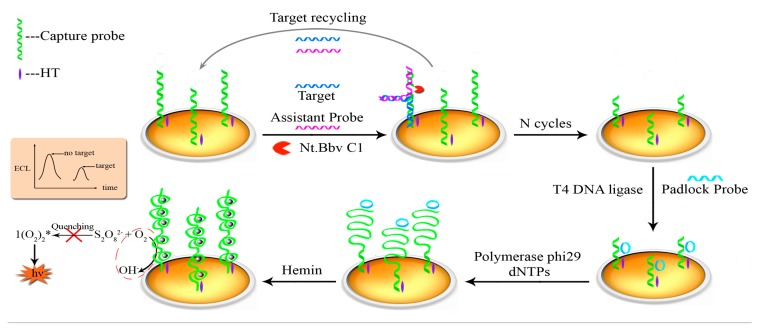
Illustration of the dual signal amplification strategy for specific DNA detection based on the Nt.BbvCI-assisted recycling amplification (TRA), rolling circle amplification (RCA) reaction and the formation of hemin/G-quadruplex.

The goal of this study is to develop a new universal and highly sensitive strategy for the ECL-meditated detection of sequence-specific DNA by coupling Nt.BbvCI-assisted TRA with an RCA reaction for signal amplification and formation of hemin/G-quadruplex. Nt.BbvCI is a nicking endonuclease that exclusively cuts one strand of a double-stranded DNA at the cleavage site XXCC▼TCAGCXX from the 5′ end to 3′ end. To make this strategy universal, we design the recognition site in the double-stranded DNA of SH-CP/AP and only the SH-CP could be cleaved. As illustrated in Figure 1, the pretreated AuE surface was modified with SH-CP via Au-S bonds and blocked with HT. Then, target DNAs hybridize with the SH-CP and AP strands to form “Y” junction structures containing specific recognition sites (in SH-CP/AP hybridization DNA) for the Nt.BbvCI enzyme. The hybridized SH-CP sequences were then cleaved into two fragments by Nt.BbvCI, leading to the release of the targeted DNA and AP due to the instability of cleaved hybridization structures. The released target DNA and AP again hybridized with other un-digested SH-CP sequences and trigger target recycling cycles, resulting in the production of numerous cleaved SH-CPs (on the electrode) with bare binding sites for the padlock probe. Padlock probes ligated with T4 DNA ligase were bound to the digested shorter SH-CPs (on the electrode) via hybridization. Upon the addition of phi29 polymerase and dNTPs, the RCA reaction was isothermally executed at 37 °C at the 3′ end of the shorter SH-CP to generate long single-stranded DNAs with tandem repeats containing G-quadruplex sequences. Finally, hemin/G-quadruplex were formed by incubation with hemin, which led to the consumption of oxygen and resulted in a decrease in ECL emission in the detection buffer. The decrease in ECL intensity can thus be related to the quantity of target DNA in testing samples.

### 3.2. Verification of Nt.BbvCI-Assisted TRA

**Figure 2.**
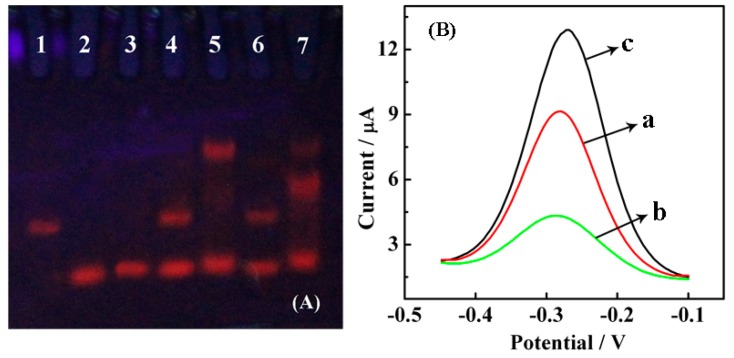
Verification of Nt.BbvCI-assisted catalyzed TRA. (**A**) Polyacrylamide gel electrophoresis: 1. SH-CP; 2. AP; 3. target DNA; 4. SH-CP/AP; 5. SH-CP/AP/target DNA (“Y” junction is observed, which is closer to the notch); 6. SH-CP/ AP/ Nt.BbvCI; 7. SH-CP/AP/target DNA/Nt.BbvCI (most of the “Y” junction is digested by Nt.BbvCI compared with lane 5). The concentrations of SH-CP, AP, target DNA and Nt.BbvCI were 5.0 μM, 5.0 μM, 5.0 μM and 5.0 U, respectively, for the above samples, which were incubated for 2 h before transferring into the gel; (**B**) DPV curves for (a) HT/SH-CP/AuE; (b) Nt.BbvCI/target DNA/AP/HT/SH-CP/AuE; (c) target DNA/AP/HT/SH-CP/AuE, the concentrations are 1.0 μM AP, 10.0 fM target DNA and 5.0 U Nt.BbvCI, incubation in 10.0 mM Tris-HCl containing 10.0 mM RuHex (pH 7.4) with a pulse amplitude of 50 mV and a pulse width of 0.05 s, respectively.

The feasibility of Nt.BbvCI-assisted TRA was explored by polyacrylamide gel electrophoresis. As observed in Figure 2A, the distinct bands in lanes 1, 2 and 3 correspond to SH-CP, AP and target, respectively. The mixture of SH-CP and AP shows two separate bands (lane 4), indicating that SH-CP and AP are stable when mixed together without any hybridization event. However, once a target is introduced, it hybridizes with SH-CP and AP to form a “Y” junction structure; thus, a UV band located closer to the notch is observed (lane 5). There is no significant difference between lanes 4 and 6, which further demonstrates that AP and SH-CP cannot form a duplex, and Nt.BbvCI does not function in the absence of a target. Only in the presence of a target can “Y” junction structures and specific recognition sites for Nt.BbvCI be formed. In lane 7, as expected, the brightness of the band at the “Y” junction structure location became dim, and a new band near the “Y” junction appeared, suggesting a lower molecular weight product forms due to digestion by Nt.BbvCI. Thus, the polyacrylamide gel electrophoresis shows that Nt.BbvCI could effectively degrade the “Y” junction structures.

The Nt.BbvCI-assisted TRA was further verified by detecting the DPV responses of RuHex at different modified surfaces. Figure 2B shows the DPV curves for modified electrodes at different stages upon TRA. Three peaks with surface-confined RuHex were recorded at approximately −0.27 V, which corresponded to the EC reduction of RuHex. The curve a represents the background current caused by the adsorption of RuHex on the SH-CP-assembled electrode surface via electrostatic interactions between the cationic RuHex and anionic phosphate DNA backbones. The hybridization between SH-CP, AP and the target DNA causes an increase in current response because of the accumulation of more RuHex on the dsDNAs (curve c). A considerable decrease in current response (curve b) was observed after the addition of Nt.BbvCI due to the recycling cleavage at the recognition site, leading to reductive and shorter anionic DNA strands localized at the electrode surface to absorb less cationic RuHex.

### 3.3. Verification of RCA and Formation of Hemin/G-Quadruplex

**Figure 3.**
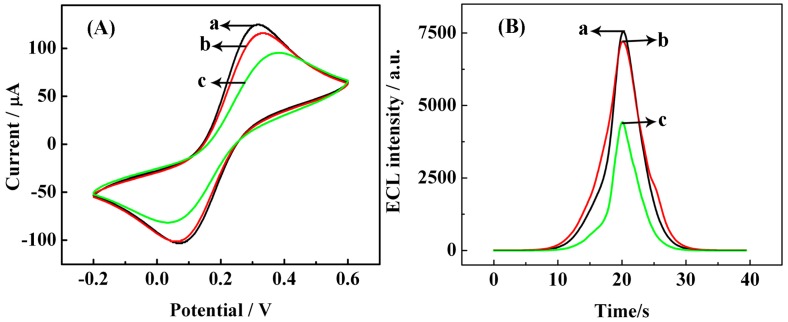
Verification of RCA and formation of hemin/G-quadruplex. (**A**) CVs at the (a) TRA/HT/SH-CP/AuE; (b) PP/TRA/HT/SH-CP/AuE; (c) RCA/PP/TRA/HT/SH-CP/AuE in the presence of 10.0 fM target DNA, were detected in 0.1 M KCl containing 1.0 mM [Fe(CN)_6_]^3−/4−^ via scanning the potential from −0.2 to 0.6 V at a scan rate of 50 mV·s^−1^; (**B**) ECL responses of differently modified electrode: (a) PP/TRA/HT/SH-CP/AuE in the presence of 10.0 fM target DNA; (b) hemin/RCA/PP/TRA/HT/SH-CP/AuE in the absence of target DNA; (c) hemin/RCA/PP/TRA/HT/SH-CP/AuE in the presence of 10.0 fM target DNA. The ECL intensity of the modified electrode was recorded in 10.0 mM PBS containing 50.0 mM K_2_S_2_O_8_ from 0 to −2 V (*vs.* Ag/AgCl) at a scan rate of 50 mV·s^−1^. The voltage of the photomultiplier tube (PMT) was set to 800 V during the process of detection.

The successful production of RCA and generation of hemin/G-quadruplex were verified by CV [34] and ECL. First, the stepwise fabrication of our proposed biosensor was characterized by CV, and the resulting cyclic voltammograms (CVs) are shown in Figure 3A. The redox couple of [Fe(CN)_6_]^3−/4−^, an indicator of surface chemistry, was used to record the electrochemical behavior of the sensor at different stages. A couple of [Fe(CN)_6_]^3−/4−^ redox peaks were observed with modified AuE, which was pretreated with SH-CP, HT, and TRA (curve a). After PP hybridizes with SH-CP, the current response (curve b) is smaller than that of curve a due to the increased negative charges on the DNA backbones. However, the current response decreased a great deal (curve c) after RCA, which was ascribed to the longer single strands on the electrode surface repelling the [Fe(CN)_6_]^3−/4−^ from the electrode.

To confirm the formation of hemin/G-quadruplex on the electrodes, ECL of a different pretreated electrode surface in 10.0 mM phosphate buffer (pH 7.4) containing 50.0 mM K_2_S_2_O_8_ was investigated. As observed with curve a in Figure 3B, the TRA-treated (incubation with 10.0 fM target DNA) HT/SH-CP/AuE generated a strong ECL signal. After RCA, the ECL intensity significantly decreased (curve c) due to the formation of hemin/G-quadruplex, which competitively consume dissolved oxygen in detection buffer, thus quenching the ECL emission that originated from the reaction between oxygen and S_2_O_8_^2−^ (the possible ECL mechanism is listed below according to the previous reports [35–39]). However, the ECL detection of electrodes treated by TRA, RCA and hemin without target DNA continued to slightly decrease (curve b), and this may be caused by the nonspecific absorption of hemin.

(1) $${\text{S}}_{2}{{\text{O}}_{8}}^{2-}+\text{e}\;\to \text{S}{{\text{O}}_{4}}^{\cdot -}+\text{S}{{\text{O}}_{4}}^{2-}$$

(2) $${{\text{SO}}_{4}}^{\cdot -}+{\text{H}}_{2}\text{O}\;\to {\text{HO}}^{\cdot }+{{\text{HSO}}_{4}}^{-}$$

(3) $${\text{HO}}^{\cdot }\to {\text{HOO}}^{\cdot }+{\text{H}}_{2}\text{O}$$

(4) $${\text{O}}_{2}+{\text{H}}_{2}\text{O}+\text{e}\;\to {\text{HOO}}^{\cdot }+{\text{HO}}^{-}$$

(5) SO4⋅−+HOO⋅→HSO4−+(O2)21∗

(6) (O2)21∗→23O2+hv

### 3.4. Optimization of TRA and RCA Experimental Conditions

**Figure 4.**
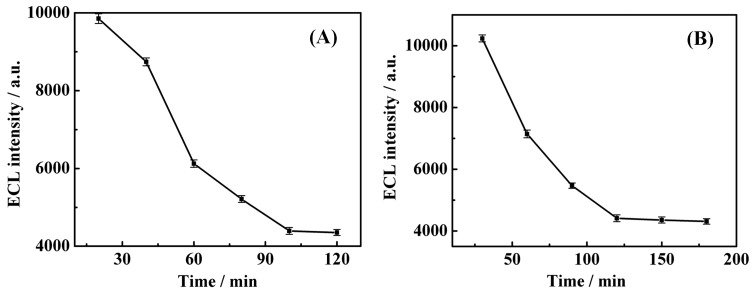
Optimization of the TRA and RCA experimental duration. (**A**) TRA duration-dependent ECL intensity; (**B**) RCA duration-dependent ECL intensity changes. The ECL measurement conditions were the same as in Figure 3B. All experiments were repeated 3 times (error bars = SD, *n* = 3).

Our strategy is primarily dependent on the Nt.BbvCI-assisted TRA reaction. Theoretically, more cleaved SH-CPs can facilitate the subsequent elongation via RCA, which finally leads to a higher quenching effect toward the O_2_/S_2_O_8_^2−^ system. The effect of TRA cleavage time on signal enhancement was investigated to detect 10.0 fM target DNA in the presence of 5.0 U Nt.BbvCI. After experiments performed with different durations of Nt.BbvCI-assisted cleavage at a constant RCA time (2 h), the ECL intensities were recorded (Figure 4A). The ECL intensity rapidly decreased with the cleavage in the TRA reaction from 0 to 100 min. However, after 100 min, the ECL response almost leveled off, indicating that the TRA reaction has reached equilibrium. Therefore, 100 min was chosen as the optimal duration for TRA in subsequent experiments.

In addition, the effect of the RCA reaction time was also evaluated by performing the experiments at constant optimal TRA reaction duration (100 min) and various RCA reaction times. As observed in Figure 4B, the amplification duration varied from 30 to 180 min with an interval of 30 min. It was found that the ECL readout rapidly decreased with the RCA reaction time up to 120 min. However, the signal exhibited no further significant decrease when the reaction duration went beyond 120 min. This finding may be attributed to the fact that, when the reaction duration was increased beyond 120 min, the RCA products were entangled with each other, which could hinder extension of RCA product. Therefore, 120 min was selected as the optimum RCA time in subsequent experiments.

### 3.5. Sensitivity and Selectivity of the ECL Biosensor

**Figure 5.**
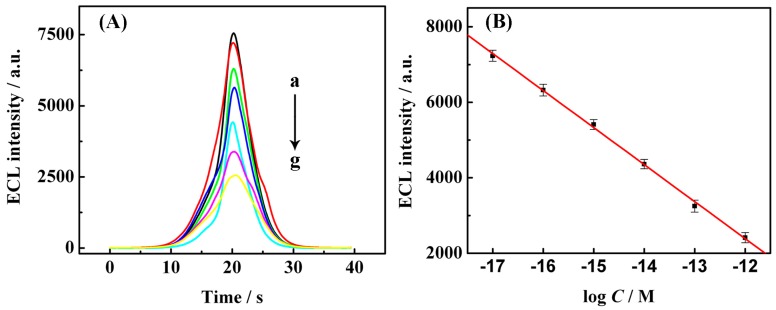
Sensitivity of modified electrodes. (**A**) ECL responses of the sensors to different concentrations of target DNA: (a) 0 M; (b) 10.0 aM; (c) 100.0 aM; (d) 1.0 fM; (e) 10.0 fM; (f) 100.0 fM; (g) 1.0 pM; (**B**) The resulting calibration plot of log c *vs.* ECL intensity (y = −9371.2 − 980.1 logC; C: concentration of target DNA(M), y: ECL intensity, *R*^2^ = 0.99). ECL measurement conditions were performed as described in Figure 3B, and all experiments were repeated 3 times (error bars = SD, *n* = 3).

Because each target DNA molecule is recycled in TRA, the RCA reaction could theoretically generate numerous tandem repeats containing G-quadruplex sequences that result in a signal that is amplified thousands of times, and our proposed genosensor is expected to provide high sensitivity with amplification of the intercalated ECL signal quencher hemin in S_2_O_8_^2−^ buffer. The ECL response to genosensors associated with different concentrations of target DNA in testing buffers serve as the basic value for target determination. As shown in Figure 5A, a relatively strong basic ECL response was obtained in the absence of target DNA. However, the addition of an increasing amount of target DNA resulted in a dynamic decrease in ECL intensity. Because more target DNA led to the generation of more hemin/G-quadruplex that could competitively consume the dissolved oxygen in detection buffer, there was a higher quenching effect toward the O_2_/S_2_O_8_^2−^ system. This strategy demonstrates a good relationship between the concentration of target DNA and ECL intensity (y= −9371.2 − 980.1 log C, C: concentration of target DNA (M), y: ECL intensity) with a good regression coefficient (*R*^2^ = 0.99). The linear range was from 10.0 aM to 1.0 pM for the target DNA (Figure 5B), and the estimated detection limit was 3.8 aM (3σ). Moreover, the coefficient of variation (CV, *n* = 6) of the target DNA at the 10.0 fM level with different electrodes from one batch was 6.5%, showing good intraassay reproducibility for the ECL sensor. Interassay reproducibility was also tested with six repetitive measurements at 10.0 fM target DNA with one electrode and a CV of 5.7% was obtained.

**Figure 6.**
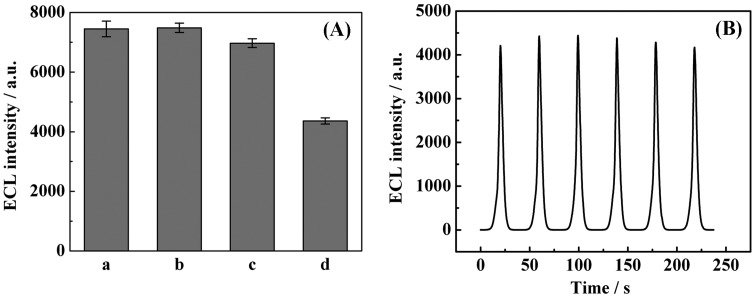
Selectivity and stability of the modified electrodes. (**A**) Selectivity for the target DNA against nDNA and sDNA was investigation: (a) blank solution (0 pM target DNA); (b) 100.0 fM nDNA; (c) 100.0 fM sDNA; and (d) 10.0 fM target DNA, the experiments were repeated 3 times (error bars = SD, *n* = 3); (**B**) ECL profiles of the sensor for 10.0 fM target DNA with 6 continuous CV cycles.

To evaluate the selectivity of the proposed genosensor, we compared the change in ECL intensity caused by the complementary (target), single-base mismatched (sDNA) and non-complementary sequence (nDNA). As shown in Figure 6A, the ECL signals for 100.0 fM sDNA (column c) and nDNA (column b)were slightly weaker than that of the control experiment (column a), which had an absence of target DNA. However, the presence of a lower (10-fold) concentration of target DNA (column d, 10.0 fM) yielded a significantly weaker ECL signal compared with the blank and excess nontarget DNA. This comparison suggests that only perfect match DNA triggers the TRA and RCA processes efficiently. Thus, the TRA and RCA system is capable of identifying complementary target DNA sequences and nontarget sequences, suggesting good selectivity for our proposed genosensor. Moreover, the stability of the proposed TRA and RCA-based DNA assay was also examined by scanning a continuous CV for 6 cycles (shown in Figure 6B), and accordingly, sharp ECL peaks with almost constant intensity was obtained, suggesting good stability of this sensor.

The recovery experiments were performed by standard addition methods in human serum to monitor the feasibility of the developed ECL genosensor. The results were shown in Table 2 and the recovery was in the range of 96.1%–103.0%, which demonstrated that our strategy could be considered as an optional scheme for detection of *S. aureus* DNA in clinical diagnostics.

## 4. Conclusions

We have demonstrated the construction of a highly sensitive ECL genosensor for detecting sequence-specific DNA based on TRA, RCA and hemin/G-quadruplex. The signal amplification relies on “Y” junction formation followed by TRA and RCA on the sensing surface and the generation of massive hemin/G-quadruplex. The proposed ECL sensor was compared with other DNA genosensors reported in literatures for the detection of DNA, as listed in Table 3. The sensors based on RCA showed lower detection limit at least one order of magnitude than most of the others, and our ECL sensor combined TRA, RCA and hemin/G-quadruplex exhibited comparable (2.6–18.7 fold-improvement) detection limit against the other two RCA-based ECL sensors [40,41].With the effective signal amplification routes, our protocol provides aM (3.8 aM) detection of target DNA. In addition, this DNA detection approach has also excellent selectivity, which is evidenced by its detection capability of single base-mismatched DNA. In this paper, we only show the capability of the proposed strategy for detecting a *S. aureus* specific DNA sequence; however, our method can be expanded to detect a wide range of DNA sequences if corresponding assistant, capture and padlock probes are specifically chosen and designed.

## Supplementary Materials

Supplementary materials can be accessed at: http://www.mdpi.com/1424-8220/15/2/2629/s1.



## Figures and Tables

**Table 1.** The oligonucleotide sequences used in our strategy.

**Note**	**Sequence(5**′–**3**′**)**
Target	AATATACGCTAAGCCACGTCCA
Capture probe (SH-CP)	SH-TTTTTTTTTTATGGACGTGGCTCCTCAGCTTT
Assistant probe (AP)	GCTGAGGAAATTAGCGTATATT
Padlock probe (PP)	pCGTCCATGTAGTACAGACGCAGTATTAGCACAAAAA CCACACGATCCTAAAAACCCAACCCGCCCTACCCGGGAGCCA *
Single base mismatchsequence (sDNA)	AATATACGCCAAGCCACGTCCA
non-complementary sequence (nDNA)	CGGTCCGATCGCCATTGCAGAC

* The underlined letters is completely complementary with the G-quadruplex sequence, and p in the padlock probe represents phosphate at the 5′ end.

**Table 2.** The recovery of the proposed ECL genosensor in normal human serum.

**Sample Number**	**Add/fM (*n*** = **5)**	**Found/fM**	**Recovery/**%
1	5	4.9 ± 0.13	98.0
2	10	10.3 ± 0.21	103.0
3	20	19.9 ± 0.36	99.5
4	50	51.2 ± 0.28	102.4
5	100	96.1 ± 0.62	96.1

**Table 3.** Comparison of the proposed electrochemiluminescent(ECL) sensor with some reported genosensors.

**Detection Technique**	**Type of Assay**	**Detection Limit**	**Signal Amplification Strategy**	**Reference**
ECL	Sandwich	15.0 fM	Ru(phen)_3_^2+^ intercalated into HCR procuct	Ying Chen *et al.* (2012) [40]
ECL	Indirect	10.0 aM	Hyperbranching RCA	Yi Long *et al.* (2011) [41]
ECL	Sandwich	1.2 fM	Magnetic streptavidin-coated nanobeads	Liping Shen *et al.* (2012) [42]
ECL	Direct	5.0 pM	Molecular beacon modified with ferrocene	Aihong Wu *et al.* (2010) [43]
ECL	Sandwich	3.0 fM	Acridinium NHS ester (AE NHS) labels attached to DNA	Yi He *et al.* (2011) [44]
ECL	Sandwich	8.5 aM	Calcium carbonate/carboxymethyl chitosan (CaCO3/CMC) hybrid microspheres @ luminescent silver nanoparticles (AgNPs) composites	Meng Li *et al.* (2014) [45]
ECL	Direct	100.0 fM	Nano-gold enhancement and ferrocene quenching	Wu Yao *et al.* (2013) [46]
Chemiluminescence	Indirect	71.0 aM	RCA	Bi Sai *et al.* (2010) [47]
ECL	Indirect	3.8 aM	TRA and RCA	This work
