# Supplementary material for: A Novel Ultrasensitive ECL Sensor for DNA Detection Based on Nicking Endonuclease-Assisted Target Recycling Amplification, Rolling Circle Amplification and Hemin/G-Quadruplex
Source: Sensors (Basel). 2015 Jan 26;15(2):2629–43. doi: 10.3390/s150202629 (PMC4367324; doi:10.3390/s150202629)
Supplement: Supplementary file 1 [file sensors-15-02629-s001.pdf]

OPEN ACCESS

sensors

ISSN 1424-8220
www.mdpi.com/journal/sensors

Supplementary Information

A Novel Ultrasensitive ECL Sensor for DNA Detection Based on Nicking Endonuclease-Assisted Target Recycling Amplification, Rolling Circle Amplification and Hemin/G-Quadruplex. *Sensors* 2015, *15*, 2629-2643

Fukang Luo †, Guimin Xiang †, Xiaoyun Pu *, Juanchun Yu, Ming Chen and Guohui Chen

Department of Clinical Laboratory, Xinqiao Hospital, Third Military Medical University, Chongqing 400037, China; E-Mails: luofukang@tmmu.edu.cn (F.L.); guimin_xiang@sina.cn (G.X.); yxyxyx_668@163.com (J.Y.); mingchen323817@163.com (M.C.); guohui_chen@sina.cn (G.C.)

† These authors contributed equally to this work.

* Author to whom correspondence should be addressed; E-Mail: xiaoyun_pu@sina.cn; Tel.: +86-23-68755637; Fax: +86-23-68755637.

1. Cylic Voltammograms (CVs) of Pretreated Gold Electrode (AuE)

AuEs were first immersed in a freshly prepared mix of concentrated sulfuric acid and 30% peroxide solution (3:1 (v/v)) for 30 min. Then, the electrodes were thoroughly rinsed with ultrapure water, polished with a 0.3 and 0.05 μm aluminum slurry and sequentially sonicated in ultrapure water, ethanol and ultrapure water for 5 min each. Afterward, the electrodes were electrochemically cleaned in 0.5 M H_2SO_4 with potential scanning from −0.3 to 1.55 V until a remarkable voltammetric peak was obtained, followed by sonication and drying with N_2. The CVs of pretreated AuEs in 0.5 M H_2SO_4 are as follows, and the remarkable voltammetric peaks (a, b) can be seen at different scan rate (1/0.1 $V{\cdot}s^{-1}$), which indicate the AuE is clean and ready to use.

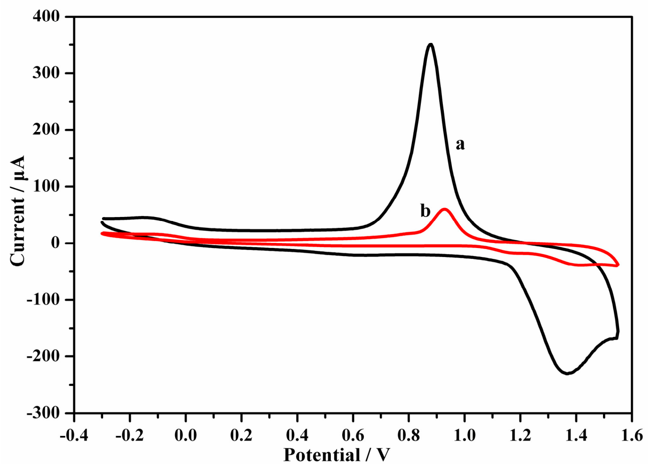

Figure S1. CVs of pretreated AuEs in 0.5 M H_2SO_4. (a) at a scan rate of 1 V·s^{-1}; (b) at a scan rate of 0.1 V·s^{-1}. CVs were detected in 0.5 M H_2SO_4 via scanning the potential from −0.3 to 1.55 V.

2. Verification of SH-CP Modified AuE

A 10.0 µL capture probe (SH-CP) (1.0 µM) droplet was cast onto a pretreated AuE and incubated overnight at room temperature. And a self-assembled monolayer (SAM) of SH-CP is formed on the electrode via Au-S bonds. To verify the SAM formed, CVs of the electrode were recorded during this process. We can see the current intensity of modified electrode (b) decreased obviously compared with unmodified one (a), that shows SAM is formed on the surface of the electrode.

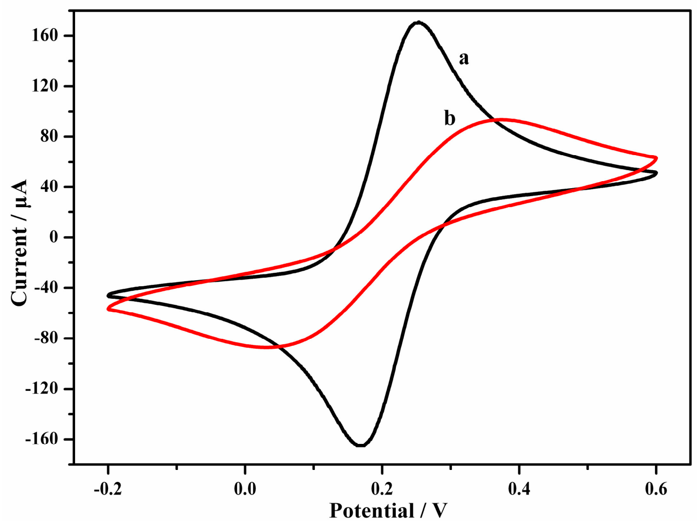

Figure S2. CVs of AuE during the modification process. (a) The pretreated AuE without SH-CP modification; (b) The AuE modified with SH-CP. CVs were detected in 0.1 M KCl containing 1.0 mM [Fe(CN)6]3-/4- via scanning the potential from −0.2 to 0.6 V at a scan rate of 50 mV·s^{-1}.

© 2015 by the authors; licensee MDPI, Basel, Switzerland. This article is an open access article distributed under the terms and conditions of the Creative Commons Attribution license (http://creativecommons.org/licenses/by/4.0/).
